# Persistence of *Listeria monocytogenes*: an integrative narrative review

**DOI:** 10.3389/fmicb.2026.1721836

**Published:** 2026-01-20

**Authors:** Raúl A. Poutou-Piñales, Sandra M. Rincón-Gamboa, Ana K. Carrascal-Camacho

**Affiliations:** 1 Laboratorio de Biotecnología Molecular, Grupo de Biotecnología Ambiental e Industrial (GBAI), Departamento de Microbiología, Facultad de Ciencias, Pontificia Universidad Javeriana, Bogotá, Colombia; 2 Laboratorio de Microbiología de Alimentos, Grupo de Biotecnología Ambiental e Industrial (GBAI), Departamento de Microbiología, Facultad de Ciencias, Pontificia Universidad Javeriana, Bogotá, Colombia

**Keywords:** environmental and genetic factors, food chain (MeSH), *Listeria monocytogenes*, persistence, reintroduction

## Abstract

The persistence of *Listeria monocytogenes* has become a public health problem in food safety due to its ability to survive in humans, animals, industrial environments, and food matrices through mechanisms of genetic, physiological, and ecological plasticity. The objective of this exploratory review was to map and synthesise scientific information published between 2000 and 2025 on the determinants that sustain this phenomenon, integrating clinical, agricultural and industrial, information from a general perspective. The findings show that a limited set of clonal complexes, including CC1, CC2, CC4, CC6, CC9, CC31, CC87, CC121, and CC321, are recurrently associated with episodes of long-term persistence. At the molecular level, genes such as *qac*H, *bcr*ABC, *cad*AC, and *mdr*L, together with stress survival islands (SSI-1 and SSI-2), provide tolerance to quaternary ammonium compounds, metals, and hostile conditions, and the truncated variants of *inl*A show adaptive trajectories that favour colonisation in food over invasion in humans. In the host, the resistance to bile and the antimicrobials favours progression to invasive infections; since the agricultural systems, soils, and livestock act as chronic reservoirs and in processing plants, the persistence becomes favoured by biofilms formation, structural and construction deficiencies in drains and some equipment, because of the difficulty in accessing all pieces and parts during cleaning. Overall, this review demonstrates that the persistence of *L. monocytogenes* results from the convergence of genetic, physiological, and environmental factors, and highlights the need for interdisciplinary and comprehensive control strategies aimed at mitigating its impact on the food chain.

## 1 Introduction

*Listeria monocytogenes* is a Gram-positive bacterium widely distributed in the environment that is crucial to public health due to its ability to cause listeriosis in humans and animals (Orsi and Wiedmann, 2016). This disease is associated with high rates of hospitalisation and mortality, particularly in vulnerable populations, as well as significant economic losses (de Noordhout et al., 2014).

Beyond its clinical impact, one of the most relevant features of *L. monocytogenes* is its ability to persist in the environment and to colonise food processing facilities. This characteristic reinforces its role as a zoonotic pathogen of global importance and represents a critical challenge for food safety (Rincón-Gamboa et al., 2024).

In Europe, persistence is a microbiological hazard, as it involves the survival of the microorganism in specific niches within production environments, despite the continuous application of cleaning and disinfection measures. However, it is essential to differentiate between persistence and the continuous reintroduction of the microorganism through raw materials or other external routes, a key distinction for correct epidemiological interpretation and the implementation of effective control strategies (Koutsoumanis et al., 2024).

From a conceptual viewpoint, persistence of *L. monocytogenes* appears to be used in several ways in the literature. In general, the term describes the repeated detection of genetically indistinguishable strains in the same food processing environment over prolonged periods, which can range from months to years or even decades (Finn et al., 2023). This phenomenon has been documented in meat, poultry, dairy and agricultural processing plants, and is associated with recurrent food contamination and foodborne outbreaks (Ferreira et al., 2014).

In this context, this article adopts an operational approach that distinguishes between persistence and continuous reintroduction, recognising that both can generate similar epidemiological patterns. Persistence occurs when genetically indistinguishable strains remain in specific niches of the processing environment over time, resulting from their adaptation to the environment (Lundén et al., 2003). In contrast, reintroduction is related to the recurrent entry of the microorganism from external sources, such as raw materials, without conclusive evidence of environmental adaptation (Koutsoumanis et al., 2024). However, this distinction is not always absolute and depends on the production context and sampling design, which reinforces the need to interpret both concepts with caution.

### 1.1 Conceptual framework of the review

Although scoping reviews do not seek to test hypotheses in the experimental or statistical sense, it is relevant to propose a guiding assumption that serves as a conceptual framework for organising the information collected. In this context, the scoping review was conducted, based on the hypothesis that:

“The persistence of *L. monocytogenes* in the food chain is not the result of a single isolated factor, but rather the dynamic interplay between genetic, physiological, ecological, and structural determinants, which allows some clones to adapt, persist, and circulate continuously between human and animal hosts, industrial environments and ready-to-eat foods.”

The previous hypothesis was not analysed statistically, as is usually the case. It was intentionally adapted as an integrating axis for recurrent patterns detection, highlighting clones and genes recurrently linked to persistence and pointing out gaps in knowledge. In this way, the hypothetical approach contributed to giving coherence to the review and to projecting lines of research and control strategies aimed at comprehensive management of the phenomenon.

### 1.2 Review objective

The purpose of this review was to map and synthesise the available scientific information on the persistence of *L. monocytogenes* in varied environments, from human and animal hosts to industrial environments and ready-to-eat foods, to identify the genetic, physiological and ecological mechanisms that underpin this phenomenon through the integration of data generated in clinical studies, primary and transformation chains, the review sought to provide a comprehensive overview of the problem, highlight the clones and molecular determinants recurrently associated with persistence, and identify knowledge gaps that should guide future lines of research and control strategies.

## 2 Methodology

This study was designed as a critical and integrative narrative review with a conceptual and interpretative nature, aimed at analysing how the concept of *L. monocytogenes* persistence has been defined and operationalised in the scientific literature in different contexts, including human health, animal production, food processing environments and end products. Rather than providing an exhaustive or systematic synthesis of all available evidence, the objective was to identify conceptual patterns, recurring methodological approaches, and interpretative tensions associated with the distinction between persistence and reintroduction, as previously discussed in the literature (Finn et al., 2023; Koutsoumanis et al., 2024).

### 2.1 Information sources and bibliographic search

The search includes high-visibility international databases (PubMed, Scopus, and Web of Science), supplemented with literature available on Google Scholar to ensure broad coverage. Combinations of keywords in English and Spanish were used, such as *L. monocytogenes*, persistence, biofilms, food processing plants, clonal complex, genomic surveillance, and ready-to-eat foods. The search strategy was deliberately broad to capture the diversity of conceptual and operational uses of the term persistence in scientific literature (Finn et al., 2023).

### 2.2 Selection criteria

Studies were selected based on their conceptual and analytical relevance to the article’s objective. Priority was for original research articles, reviews, and technical reports published between 2000 and 2025 that explicitly addressed the persistence of *L. monocytogenes*, including aspects such as prolonged detection of strains, clonal recurrence, adaptation to environmental niches, or differentiation between persistence and continuous reintroduction (Lundén et al., 2003; Koutsoumanis et al., 2024). Grey literature, theses, non-peer-reviewed reports, and untraceable publications were excluded.

### 2.3 Information selection and organisation

The selection was carried out in two phases: first by title and abstract, and then by reading the entire article. Articles that met the inclusion criteria were separately and distributed into four thematic categories: (i) persistence in the human host, (ii) persistence in animals and primary chains, (iii) persistence in processing plants, and (iv) persistence in finished products.

### 2.4 Synthesis and analysis

The information was systematised in tables and comparative narratives highlighting persistent clones, resistance genes, structural factors of the facilities, and typing methodologies (PFGE, MLST, cgMLST, wgMLST, WGS, and SNP/SNV). In agreement with the narrative and conceptual nature of the study, no formal assessment of bias risk or individual study quality was done, as the emphasis resided on the interpretative consistency and critical analysis of concepts rather than quantitative comparison of results.

## 3 State of the art

The conceptual framework of “bacterial plurality” proposed by Ehrlich et al. (2005), broadens our understanding of this phenomenon by pointing out that bacteria do not exist as clonal populations, but rather as communities organised into biofilms with high phenotypic and genotypic heterogeneity. Biofilms can exhibit antimicrobial resistance up to a 1,000 times greater than that of planktonic cells, while horizontal gene transfer mechanisms facilitate the continuous generation of adapted variants. These findings explain the ability of *L. monocytogenes* to establish itself for long periods in food industry and resist inadequate disinfection protocols, emphasising the need for specific strategies to counteract biofilm formation (Ehrlich et al., 2005).

Although numerous genes related to resistance, adhesion, stress, or virulence have been described more frequently in strains classified as persistent, the available evidence does not allow most of them to be attributed a direct causal role in environmental persistence. For this reason, Table 1 presents the genetic determinants for which there is solid experimental or associative evidence documenting the persistent detection of the clone over time in food processing environments. Other functionally plausible markers are discussed in the text as contextual facilitators, without implying direct causality.

**Table 1:** Genetic determinants with solid experimental or associative evidence in the persistence of *Listeria monocytogenes* in food processing environments.

Genetic determinants	Main function	Documented environmental context	Evidence of persistence	Ref.
*bcr*ABC	Tolerance to quaternary ammonium compounds (QAC)	Processing plants, dairy industry	Association with multi-year clonal persistence under disinfectant pressure	Cooper et al. (2021); Fagerlund et al. (2022a); Fagerlund et al. (2022b)
*qac*H / Tn6188	Resistance to QAC	Food and dairy plants	Frequent in persistent strains exposed to repeated disinfection	Fagerlund et al. (2022a); Fagerlund et al. (2022b)
*cad*AC/*cad*A1C	Cadmium resistance	Industrial environments	Association with prolonged persistence in structural niches	Lundén et al. (2003)
SSI-1	Cold, acid and salinity tolerance	Processing environments and foodstuffs	Over-represented in persistent isolates	Wang et al. (2022)
SSI-2	Alkaline and oxidative stress tolerance	Industrial plants	Associated with strains adapted to adverse environments	Cooper et al. (2021)
*mdr*L	Multidrug efflux	Processing plants	Recurrent detection in persistent strains	Fagerlund et al. (2022a)
*emr*E	Biocide tolerance	Industrial environments	Detection of recurrence in persistent strains	Fagerlund et al. (2022a)

Several characteristics of *L. monocytogenes* may be crucial to its persistence in food industries, including tolerance to disinfectants, early adhesion, formation of complex biofilms, and the presence of mobile genetic elements such as plasmids and prophages (Wang et al., 2022; Rohilla et al., 2024). WGS allowed a close approach in the persistence isolates study, facilitating comparison between SNP resolution and wgMLST (Sullivan et al., 2022). Some clones of lineage II (CC121) have been frequently more associated with persistence. In contrast, those of lineage I, although more virulent, have a lower ability to establish themselves in processing environments (van de Merwe et al., 2024), most likely because they require a higher eukaryotic host to survive and multiply easily.

The use of WGS has improved the ability to compare *L. monocytogenes* isolates and assess their genetic relationship in different contexts. However, the application of WGS has not eliminated conceptual ambiguity in distinguishing between persistence and continuous reintroduction. As recent reviews pointed out, studies differ widely in the SNP thresholds used, the duration of temporal sampling, and the epidemiological interpretation of genomic proximity (Finn et al., 2023; Koutsoumanis et al., 2024).

In operational terms, several studies have found that isolates with low genomic differences, often within narrow SNP ranges, may be indicative of persistence when detected repeatedly in the same environment over time. However, these thresholds are not universal and must be analysed in conjunction with temporal and contextual evidence. The detection of genetically close isolates over short periods may reflect recent contamination or reintroduction events, while the recurrent recovery of closely related strains over months or years, combined with the identification of stable environmental niches, supports the interpretation of persistence (Finn et al., 2023).

Therefore, this article adopts an integrative approach in which persistence is not defined exclusively by a specific genomic threshold, but rather by the convergence of three elements: (i) high genomic similarity between isolates, assessed by WGS; (ii) documented temporal recurrence; and (iii) contextual evidence supporting the adaptation of the microorganism to specific niches in the production environment. This perspective is consistent with the need, highlighted in the literature, to avoid simplistic interpretations based solely on genomic metrics and to consider sampling design and epidemiological context in the persistence definition (Koutsoumanis et al., 2024).

The application of WGS tools is not uniform globally. In many low- and middle-income countries, limitations in infrastructure, access to molecular diagnostics, technical training, and economic resources restrict the routine use of WGS in surveillance systems. Additionally, barriers to the exchange and standardisation of genomic data make comparisons between regions difficult. In this context, the lower availability of genomic information should not be understood as a lower occurrence of persistence, but rather as a structural limitation in the generation and access to data.

Biofilm formation is a key mechanism. The roughness and topography of the surface (type and condition) of some materials, such as rubber, facilitate adhesion and confer tolerance to disinfectants. The presence of oxides on the surface of tables and equipment is another factor to consider (Assisi et al., 2021). Factors that favour the formation of Listeria biofilms include: flagellum-mediated adhesion, physical forces including Van der Waals forces, hydrophobic interactions and electrostatic forces (Møretrø and Langsrud, 2004), as well as interactions with the food plant’s own microbiota (Palaiodimou et al., 2021). The formation of these biofilms depends on the amount and type of water. Hard water with traces of magnesium and calcium salts, which can be on the surfaces, allows rough layers to form, facilitating adhesion during production operations. Various studies have established that acidic conditions generated by the use of citric acid (widely used as an acidulant) or lactic acid (used as an antimicrobial) promote the adhesion of *L. monocytogenes* Scott A to stainless steel, due to the protonation of negative groups on the bacterial surface (Briandet et al., 1999a; Briandet et al., 1999b). In mixed biofilms, where different bacterial species coexist, an increase in survival has been recorded, reflecting the influence of microbial ecology (Estrada et al., 2020) (Table 1).

From a genomic perspective, several determinants have been frequently associated with the persistence of *L. monocytogenes*, particularly in food processing environments. These include the *bcr*ABC cassette, which is associated with resistance to quaternary ammonium compounds and detected in more than 59% of environmental isolates (Cooper et al., 2021), as well as the SSI-1 and SSI-2 genomic islands and the Tn6188 transposon, which have been widely reported in large-scale WGS-based genomic studies as contributors to tolerance to adverse environmental conditions (Pasquali et al., 2018; Wang et al., 2022). Functional evidence derived from smaller-scale studies further supports the role of SSI-1, SSI-2, and Tn6188 in stress tolerance and biofilm formation under industrial conditions (Manso et al., 2019). Likewise, genes such as *bsh*, *pva*, and *btl*B, associated with bile salt tolerance, have been described. Similarly, the ClpL factor, an AAA^+^ disaggregase (E.C. 3.6.3.-), confers remarkable thermal resistance (Bohl et al., 2023).

In transcriptomic studies, persistent isolates exhibit higher expression of operons such as *pdu*, *cob*-*cbi*, and *eut*, which are related to energy metabolism and stress tolerance, potentially conferring competitive advantages in environments not associated with the host (Fox et al., 2011b). In contrast, WGS-based genomic analyses in processing environments suggest that there is no single genetic mechanism that explains persistence; persistent strains tend to harbour traits associated with hypovirulence (e.g., truncated variants of *inl*A) more frequently, while markers of resistance to disinfectants such as *qac*H can occur in both persistent and non-persistent isolates (Palaiodimou et al., 2021).

This knowledge highlights the need to integrate genomic surveillance with innovative control measures that go beyond conventional cleaning and disinfection protocols. Emerging strategies such as high-pressure processing (HPP), the use of bacteriophages and hygienic equipment design represent promising alternatives for mitigating the persistence of this pathogen and reducing its impact on the food chain (van de Merwe et al., 2024); as well as the use of different microbiological and molecular techniques that facilitate the identification and monitoring of potentially persistent isolates (Supplementary material S1).

In this regard, technologies such as bacteriophages, high-pressure processing (HPP) and improvements in hygienic design are part of the existing technical context, and they will not be addressed as control strategies in themselves, since their application in real plants depends on structural, operational and economic barriers that limit or favour their impact on persistence phenomena.

From an experimental perspective, several techniques have served to characterise persistence. Biofilm assays with crystal violet and adhesion tests (on stainless-steel surface) have shown persistent strains with a big colonisation capacity (Rohilla et al., 2024). MIC and MBC studies confirmed high tolerance to biocides (Cherifi et al., 2020), while resistance tests to cadmium (Cd^2+^), arsenic (As), and copper (Cu^2+^) linked this phenotype to genes such as *cad*A1C (Ikhimiukor et al., 2024). Microbiological methods such as selective media cultures (Oxford, PALCAM) and CFU (Colony Forming Unit) and MPN (Most Probable Number) enumeration remain indispensable for environmental detection (Domingues et al., 2025). In addition, technologies such as High Pressure Processing (HPP) have been applied to persistent lineages, revealing differences in survival between lineages I and II (van de Merwe et al., 2024).

Similarly, growth assays monitored by OD_600_ have confirmed the ability to multiply under stressful conditions, while specific tests for cold, acidity and salt tolerance have shown that persistent strains remain viable in environments that inhibit other bacteria (Taylor and Stasiewicz, 2019; Domingues et al., 2025). Protein characterisation using SDS-PAGE and MALDI-TOF MS rapid identification served to differentiate strains adapted to dairy matrices from those of sporadic origin (Ortiz et al., 2016), (Supplementary material S1).

Molecular studies have provided greater detail. PCR and multiplex PCR techniques have enabled the detection of internalins such as *inl*A or *inl*B responsible for adhesion to epithelial cells. Truncated variants of *inl*A are associated with clones (CC8, CC9, CC31, and CC121), which are better adapted to food than to human hosts (Demaitre et al., 2021). PFGE was used for years to identify persistent clones (CC2, CC4, CC9, and CC121) in cheese and meat products, while more recent approaches such as MLST, cgMLST, and WGS have provided resolution at the SNP and SNV level, confirming that some clones (CC8, CC9, and CC121) are recurrently detected throughout the production and distribution chain (Cardozo-Bernal et al., 2013; Ikhimiukor et al., 2024). The use of GWAS, with genomic annotations with RAST and SPAdes assemblies, has identified accessory genes such as *bcr*ABC, *qac*H and *cad*AC, associated with tolerance to disinfectants and metals, which “likely” confer advantages in food matrices (Daeschel et al., 2022) (Supplementary material S1).

Bioinformatic techniques complement this view. Pan-genome analysis has made it possible to distinguish between “core” and “accessory” genes, identifying those enriched in persistent strains (Pasquali et al., 2018). Phylogenetic reconstruction studies based on SNPs and MLST have traced clonal evolution in several facilities (Sullivan et al., 2022). Programmes such as Prokka and RAST, together with the SPAdes assembler, have facilitated genome annotation and the identification of stress islands (SSI-1, SSI-2) related to environmental tolerance and resistance genes such as *cad*AC and *mdr*L (Daeschel et al., 2022; Domingues et al., 2025) (Supplementary material S1).

### 3.1 Persistence of *Listeria monocytogenes* in the food production chain

#### 3.1.1 Primary production environments

The persistence of *L. monocytogenes* in animals and agricultural environments is a key link in the chain of transmission to the food industry. In Canada, Grewal et al. (2007) demonstrated that the bacterium could survive in agricultural soils fertilised with contaminated cattle manure, maintaining detectable levels for months despite a progressive decline in population. To this end, microbiological methods such as culture in selective media (Oxford, PALCAM), MPN calculation and CFU counts are useful to quantify the viability of isolates under various environmental conditions. These findings highlighted the role of cattle manure and moist soil as natural reservoirs that favour the persistence of the pathogen in production systems.

On dairy farms, the occurrence and persistence of genotypes have been documented for more than 3 years on milking surfaces and drinking troughs (Castro et al., 2018). In South America, clones [CC9 (ST9), CC121 (ST121)] can persist in plants for up to 9 years (Camargo et al., 2019). In farm soils, the survival of *L. monocytogenes* has been 60 to 90 days under controlled conditions, with loamy soils mixed with compost being more favourable than sandy soils (Alegbeleye and Sant’Ana, 2022). Some strains are responsible for persistence in milking equipment, due to their greater ability to adhere and form biofilms compared to sporadic strains (Latorre et al., 2011).

The proximity between farm environments (soil, manure, animal feed) and artisanal processing plants favours the transmission of Listeria, sharing identical genomic clusters between both contexts, which demonstrates that farms act as primary reservoirs (Bolten et al., 2024). In agricultural environments, the use of mulch modifies the soil microclimate, prolonging persistence compared to uncovered soils (Micallef et al., 2023). In contrast, in hydroponic systems, survival is limited to periods of 1 to 14 days (Dhulappanavar and Gibson, 2023).

Irrigation water is a key reservoir; its persistence depends on the season, surrounding land use, and the presence of animals, which increases the risk of fresh water contamination (Gartley et al., 2022).

#### 3.1.2 Persistence in food processing plants

*Listeria monocytogenes* persistence in processing plants has been documented as a recurring phenomenon, in which specific clones survive intensive cleaning and disinfection, establishing themselves in hard-to-reach niches such as drains, damp surfaces and stainless-steel equipment. In Finland, (by using the PFGE) T, D, and F profiles of serotypes 1/2a, 1/2b, and 4b showed persistence for years in meat and fish facilities (Lundén et al., 2000; Lundén et al., 2003). Subsequent studies using MLST, cgMLST, wgMLST, and WGS confirmed the low genetic diversity of these strains, which allowed them to be differentiated from sporadic reintroductions, as the clones found (CC4, CC6, CC8, CC9, CC31, and CC121) were persistent (Latorre et al., 2011; Sullivan et al., 2022).

It is important to note that the recurrence of these clonal complexes does not imply homogeneous mechanisms of persistence, since while clones belonging to lineage II (e.g., CC9, CC121, and CC321) have been consistently related with environmental adaptation in processing plants, several clones from lineage I (such as CC4 or CC6) are predominantly associated with hypervirulence and clinical cases, reflecting different selective pressures depending on the context.

**Figure 1:**
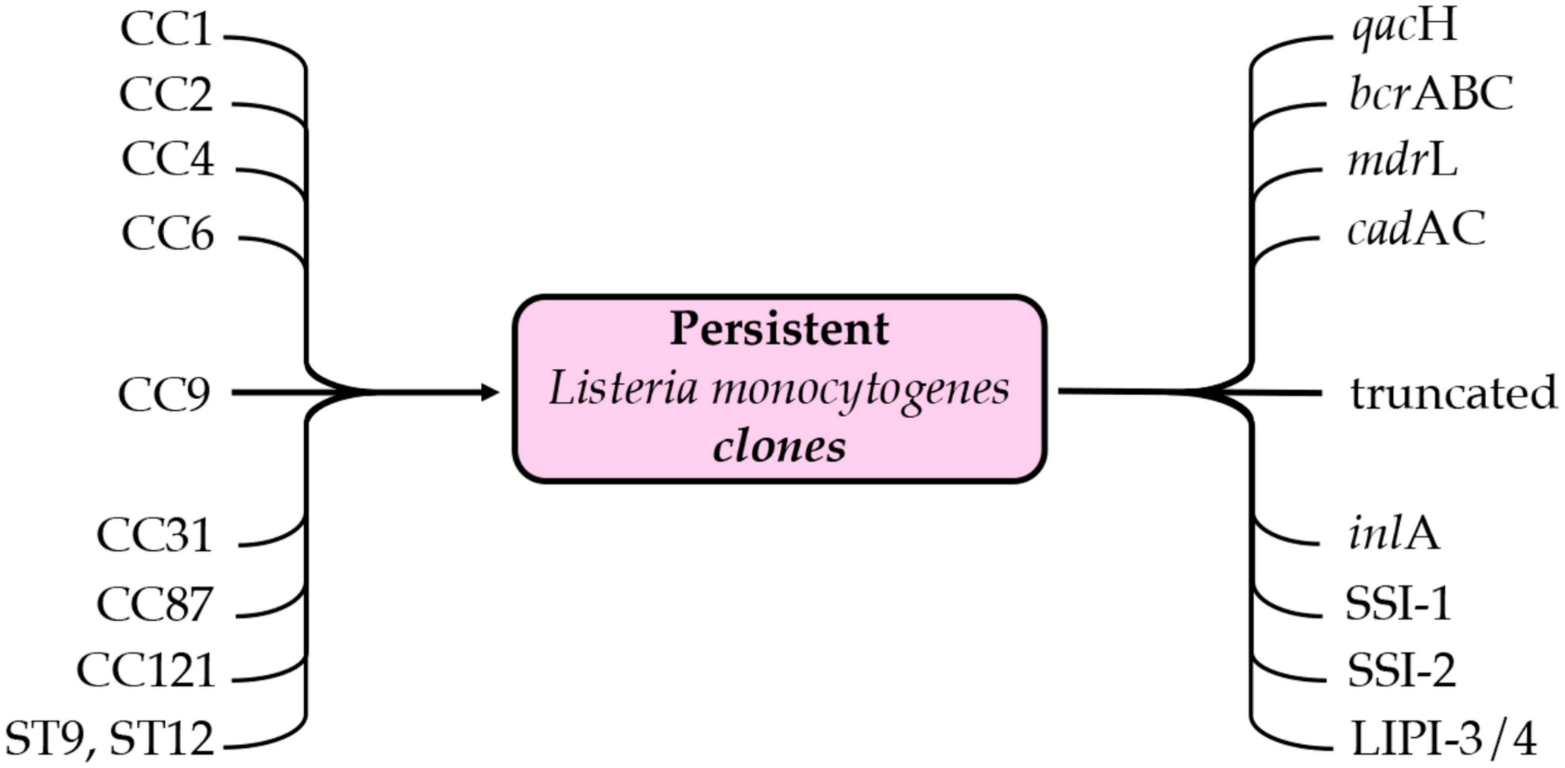
Persistent *Listeria monocytogenes* clones (CC1, CC2, CC4, CC6, CC9, CC31, CC87, CC121, CC321, ST9, and ST121) and their main genetic determinants associated with persistence and adaptation, including resistance genes (*qacH*, *bcrABC*, *mdrL*, *cadAC*), stress survival islands (SSI-1, SSI-2), truncated *inlA* variants, and pathogenicity islands (LIPI-3/4).

In these industrial environments, genetic determinants play a very crucial role. Genes such as *bcr*ABC and *qac*H, associated with tolerance to quaternary ammonium compounds, are present in persistent clones (CC9, CC121, and CC321) (Daeschel et al., 2022). Similarly, *sig*B product coordinates the general stress response and regulates genes involved in biofilms, while *prf*A product interconnects mechanisms of virulence and environmental persistence (Begley et al., 2005). Other genes, such as *bap*L (adhesion) and the teichoic acid modifiers *gtc*A and *rml*T, products strengthen adhesion to abiotic surfaces (Cherifi et al., 2020). In addition to these, there are the LIPI-3 and LIPI-4 (pathogenicity islands) and the metal resistance genes such as *cad*AC, which provide additional advantages in industrial environments (Ikhimiukor et al., 2024) (Figure 1). The identification of plasmids (pLM1686, pLM1692) and prophages in loci such as comK have shown that mobile elements amplify genetic plasticity and ensure survival (Wang et al., 2022).

In Colombia, a study of meat processing plants (from the department of Boyacá) found potentially persistent isolates on equipment in direct contact with food, such as slicers, knives, and cutting boards. These sites, used during the packaging phase, were identified as niches and risk points, as the product did not undergo further treatment. Serotypes 4b, 1/2a, and 1/2c were identified, with dissemination observed in several samples, suggesting adaptation of the bacterium in production environments. However, persistence confirmation requires sequencing studies to establish clonal similarity (Rincón-Gamboa et al., 2024).

The Colombian findings coincided with those from Europe and North America when pointing out that equipment such as saws, among others, acted as possible reservoirs. Similarly, the ability of *L. monocytogenes* to resist low temperatures, incomplete cleaning processes, acid and saline environments was due to the formation of biofilms. Control measures recommended included strengthening and implementing environmental pathogen monitoring programmes, updating cleaning protocols, and using enzymatic agents to remove biofilms, as well as correlating environmental isolates with clinical cases of listeriosis in humans (Rincón-Gamboa et al., 2024).

#### 3.1.3 Persistence in finished products

The persistence of *L. monocytogenes* in ready-to-eat foods poses a critical public health risk, as these products are consumed without additional processes to eliminate the pathogen. In Ireland, Fox et al. (2011a, 2011b) experimentally demonstrated that in inoculated soft cheeses, the bacteria can not only survive throughout the shelf life, but also multiply under refrigerated conditions, confirming the importance of factors such as pH, water activity, and milk matrix composition (Fox et al., 2011a). Subsequently, Ortiz et al. (2016) compiled information from studies in Europe and Latin America documenting the recurrent detection of the pathogen in fresh, soft and semi-hard cheeses, even in facilities with high hygiene standards.

Understanding these phenomena has been enhanced by the application of multiple techniques. Classical microbiological methods, such as CFU counts, MPN quantification and selective media (Oxford, PALCAM) (Gunasinghe et al., 1994; Capita et al., 2001) and chromogens (CHROMagar™) (Carozzi, 2005), have made it possible to detect the bacteria even at very low concentrations (Grewal et al., 2007; Cherifi et al., 2020). Growth assays monitored by OD_600nm_ confirmed the multiplicity under stress conditions, while specific tests for cold, acidity and salt tolerance have shown that persistent strains remain viable in environments that inhibit other bacteria (Domingues et al., 2025). Protein characterisation by SDS-PAGE and rapid identification by MALDI-TOF MS has allowed the differentiation of strains adapted to dairy matrices from those of sporadic origin (Ortiz et al., 2016).

Chemical tests have been equally relevant. The determination of MIC and MBC in isolates from soft cheeses have shown that persistent isolates can tolerate higher concentrations of disinfectants than non-persistent ones (Wang et al., 2015). Similarly, metal tolerance assays revealed that genes products such as *cad*A1C and *cad*AC confer advantages against cadmium and copper present in manufacturing processes (Ikhimiukor et al., 2024). The evaluation of biofilm formation capacity through biofilm assays and adhesion to stainless steel through adhesion assays have shown that isolates adapted to dairy products exhibit greater adherence to processing equipment, which facilitates the recontamination of finished products (Rohilla et al., 2024).

From the genetic perspective, in addition to *inl*A and *inl*B genes products, other such as *sig*B and *prf*A coordinate responses to stress and virulence that facilitate survival in refrigerated products (Begley et al., 2005). Intracellular genes such as *act*A, as well as adhesins (*bap*L, *atl*/p60/*ami*), expand the repertoire of adaptation factors, while antimicrobial resistance determinants (*mdr*, *fos*X, *nor*B, *sul*, *tet*M, *tet*S, *lin*) ensure tolerance to compounds present in food production (Daeschel et al., 2022). The presence of prophages and plasmids carrying resistance genes adds plasticity to the genome, reinforcing adaptation in dairy matrices (Wang et al., 2022).

Overall, studies on finished products confirm that the persistence of *L. monocytogenes* is not limited to its survival in cheese and meat products, but reflects an adaptation strategy based on genetic resistance, metabolic plasticity and the ability to colonise complex matrices (Fox et al., 2011a; Ortiz et al., 2016). The integration of microbiological, molecular, chemical, and bioinformatic methodologies has demonstrated that ready-to-eat foods represent a key scenario for the expression of bacterial persistence, with direct implications for food safety and the prevention of listeriosis outbreaks.

#### 3.1.4 Some cases of persistence by productive sector

In cheese factories, serotype 1/2a strains have been maintained in facilities for years, associated with ripening environments such as Gorgonzola cheese (Lomonaco et al., 2009). In Ireland, pulsotypes persisting for more than 6 months were also in dozens of small plants; some isolates agreed with clinical ones (Leong et al., 2017). In artisanal dairies, transmission from the agricultural environment to the plant and persistence in both contexts have been demonstrated (Bolten et al., 2024). In Switzerland, a human outbreak of listeriosis involved several isolates of serotype 4b ST6, whose tracking pointed to a cheese production plant. Authors found that surfaces were responsible for the environmental contamination of the cheese (Nuesch-Inderbinen et al., 2021).

In a poultry plant in Spain, the prevalence of *L. monocytogenes* was approximately 83.3% in deboning areas, with the ST9 and ST121 clones persisting for an entire year, highlighting the negative impact of structural and hygienic failures (Melero et al., 2019). In fruit packinghouses in the United States, the focus of EMPs is on drains and wet areas to avoid contamination from aerosol production (Estrada et al., 2020).

In marine products, such as shrimp, persistence has been linked to the cold chain and prolonged storage. The use of high hydrostatic pressures and radiation has been shown to complement conventional refrigeration in reducing the microbial load (Norhana et al., 2010). In Norway, pervasive clones such as CC121 and CC9 stay chronically maintained in fish and meat plants, associated with resistance to metals and biocides (Fagerlund et al., 2022b). In salmon and RTE product plants, strains persisted for up to 13 years, demonstrating high tolerance to disinfectants, a rapid biofilm formation and the risk of chronic contamination (Fagerlund et al., 2021).

In Brazil, WGS analysis confirmed that specific clones (L1-SL315-ST520-CT4429 and L2-SL9-ST9-CT4420) persisted for several years in different facilities, demonstrating the genomic stability of some strains in hostile environments (Camargo et al., 2019). In Shanghai, clones such as ST5 and ST121 exhibited genetic adaptations (*clp*L, *mdr*L, *lde*, SSI-1/2) and formed biofilms resistant to chlorinated disinfectants, generating adaptive advantages (Liu et al., 2022). In Denmark, clones such as ST8 and ST121 were detected in food processing plants over several years, with less than 10 SNPs of difference between isolates, confirming chronic persistence (Takeuchi-Storm et al., 2023). In Iberian ham production plants, clones resistant to quaternary ammonium compounds identified were ST31 and ST121, both containing the Tn6188 transposon, which reinforces the fact of the selective pressure of these disinfectants (Ortiz et al., 2016).

Finally, emerging strategies such as high-pressure processing (HPP, 400–600 MPa), the use of bacteriophages, and the hygienic equipment design mean several promising alternatives for mitigating the persistence of this pathogen and reducing its impact on the food chain (Sullivan et al., 2022; van de Merwe et al., 2024). However, it is necessary to implement monitoring techniques in conjunction with innovative control measures that go beyond conventional cleaning and disinfection protocols.

However, the recurrence of clones or similar genetic determinants in different countries does not imply homogeneous patterns of persistence, as the behaviour of *L. monocytogenes* depends on the interaction between the environment, operational practices and the history of each facility.

### 3.2 Is it correct to talk of *Listeria monocytogenes* persistence in humans and animals? Who knows for sure?

#### 3.2.1 “Persistence in humans”

The persistence of *L. monocytogenes* in the human host reflects the pathogen’s ability to withstand adverse conditions in the gastrointestinal tract and sustain prolonged colonisation. This phenomenon’s support is due to the genetic and physiological network that allows it to overcome gastric acidity, the detergent action of bile salts, and the pressure exerted by antibiotics and antimicrobial compounds. In Ireland, Begley et al. (2005) demonstrated that mutants with gene deletions such as *bsh* (responsible for the hydrolysis of conjugated bile salts), *btl*B (responsible for ensuring cellular tolerance to detergent compounds) and *pva* (linked to resistance to penicillin V) are essential for intestinal survival. The regulation of these determinants depends on sigB, an alternative sigma factor that coordinates the stress response, as well as prfA, a master regulator of virulence, confirming the close connection between persistence mechanisms and systemic virulence (Ehrlich et al., 2005).

At the genetic level, persistence can be explained by a broad repertoire: internalins (*inl*A, *inl*B, *inl*C, *inl*L) facilitate adhesion to epithelial cells; actA ensures intracellular dissemination; adhesins (bapL, atl/p60/ami) reinforce colonisation; and global regulators (sigB, prfA) coordinate the stress response. Antimicrobial resistance genes (*mdr*, *fos*X, *nor*B, *sul*, *tet*M, *tet*S, *lin*) increase tolerance to clinically used antibiotics (Daeschel et al., 2022). The presence of prophages (*φcom*K, *φrum*A) and plasmids carrying resistance determinants adds plasticity to the genome, ensuring adaptability (Wang et al., 2022).

In the chemical field, MIC and MBC determinations in clinical strains have shown resistance to antibiotics and biocides (Cherifi et al., 2020), while efflux pump inhibitor assays confirmed the involvement of pumps encoded by *bcr*ABC or *qac*H (Daeschel et al., 2022). Tolerance assays for metals such as cadmium, copper, and arsenic confirmed the role of genes such as *cad*A1C and *cad*AC in persistence in the host (Wang et al., 2022; Ikhimiukor et al., 2024).

Overall, the persistence of *L. monocytogenes* in humans cannot be attributed to a single mechanism, but rather to an integrated network of genes, mobile elements and global regulators expressed under conditions of intestinal stress. The combination of molecular, microbiological, chemical and bioinformatic methodologies has demonstrated that the species acts as a dynamic system, in which genetic diversity, inducible resistance and metabolic plasticity ensure prolonged persistence in the human host (Begley et al., 2005; Ehrlich et al., 2005).

#### 3.2.2 “Persistence in animals”

In Spain, Latorre et al. (2011) evaluated the presence of *L. monocytogenes* in dairy herds, analysing both faeces and raw milk. Through PCR and molecular typing using PFGE, identical pulsotypes (T, D, and F) were identified that remained in the herds for several months (Latorre et al., 2011). Genetic analysis revealed low diversity among the isolates, suggesting the stable circulation of some lineages. Subsequent research using MLST, cgMLST, WGS, and SNP and SNV analysis confirmed that closely related clones (CC1, CC2, CC4, CC6, CC8, CC9, CC31, and CC121) can be transmitted sustainably between animals and remain on farms for years, while the use of diversity indices such as Simpson’s Index of Diversity (SID) allowed differentiation between the time of the detection of microorganisms (Sullivan et al., 2022).

Although these studies focused on epidemiological aspects, it is well known that genes such as *sig*B and *prf*A reinforce the ability to resist intestinal conditions in cattle, while internalins, like *inl*A and *inl*B, facilitate mucosal colonisation (Begley et al., 2005). Resistance genes such as *mdr*L or *cad*AC confer advantages against antibiotics and metals in the agricultural environment; in this sense, the clone (CC87) isolated from animals has been the most frequently encountered. Additionally, the presence of prophages inserted into loci such as comK is associated with the adaptation of strains to specific intestinal niches (Wang et al., 2022).

From an experimental point of view, adhesion and biofilm tests on livestock materials (plastics and stainless steel) have shown that persistent strains recovered from animals have a greater capacity to colonise surfaces, which facilitates their spread to milk and the milking environment (Wang et al., 2015; Rohilla et al., 2024). Protein analysis using SDS-PAGE and rapid characterisation by MALDI-TOF MS allowed discriminating phenotypic differences between persistent and transient clones (ST31 and ST121) (Ortiz et al., 2016). In addition, resistance studies conducted through MIC and MBC determination confirmed that some animal-derived lineages can tolerate high concentrations of antibiotics and disinfectants (Cherifi et al., 2020).

The persistence of *L. monocytogenes* in animals is a mixture of epidemiological and genetic factors. Although early studies focused on the detection and typing of clones, the integration of microbiological, molecular, chemical, and bioinformatic tools have shown that livestock and their environment act as dynamic reservoirs in which strains with resistance, metabolic plasticity, and prolonged colonisation capacity become selected. This scenario justifies why animals have become an essential epidemiological bridge between natural environments, processing plants and finished foods (Grewal et al., 2007; Latorre et al., 2011).

The persistence of *L. monocytogenes* is not a single mechanism. Protective biofilms, resistance genes, failures in hygiene infrastructure, environmental factors, and intracellular reservoirs all combine in this phenomenon (Kortebi et al., 2017; Bagatella et al., 2025). Large-scale genomic surveillance studies in North America and Europe have shown that some isolates can persist for decades and are recurrently associated with listeriosis outbreaks (Unrath et al., 2021; Tuytschaever et al., 2023).

### 3.3 Integrative reflections and general considerations on the persistence of *Listeria monocytogenes*

Persistence of *L. monocytogenes* strains should not be analysed from a single perspective. In the human host, persistence is due to complex genetic networks that allow it to resist physiological defences, while in animals, it manifests itself in the ability to remain in herds and in the agricultural environment as a natural reservoir. In processing plants, the interaction between microbial factors and structural deficiencies turns the facilities into veritable niches of permanence, and in finished foods, the characteristics of the milk matrix ensure an environment that favours bacterial viability.

The review of different persistence scenarios allows us to establish that *L. monocytogenes* has developed convergent strategies to remain in multiple environments. Whether through specific genes that ensure survival in the intestine, through silent colonisation of farm animals, through adaptation to equipment and drains in processing plants, or by exploiting the properties of ready-to-eat cheeses and dairy products, the result is always the same: a remarkable ability to remain active in the food chain.

**Figure 2:**
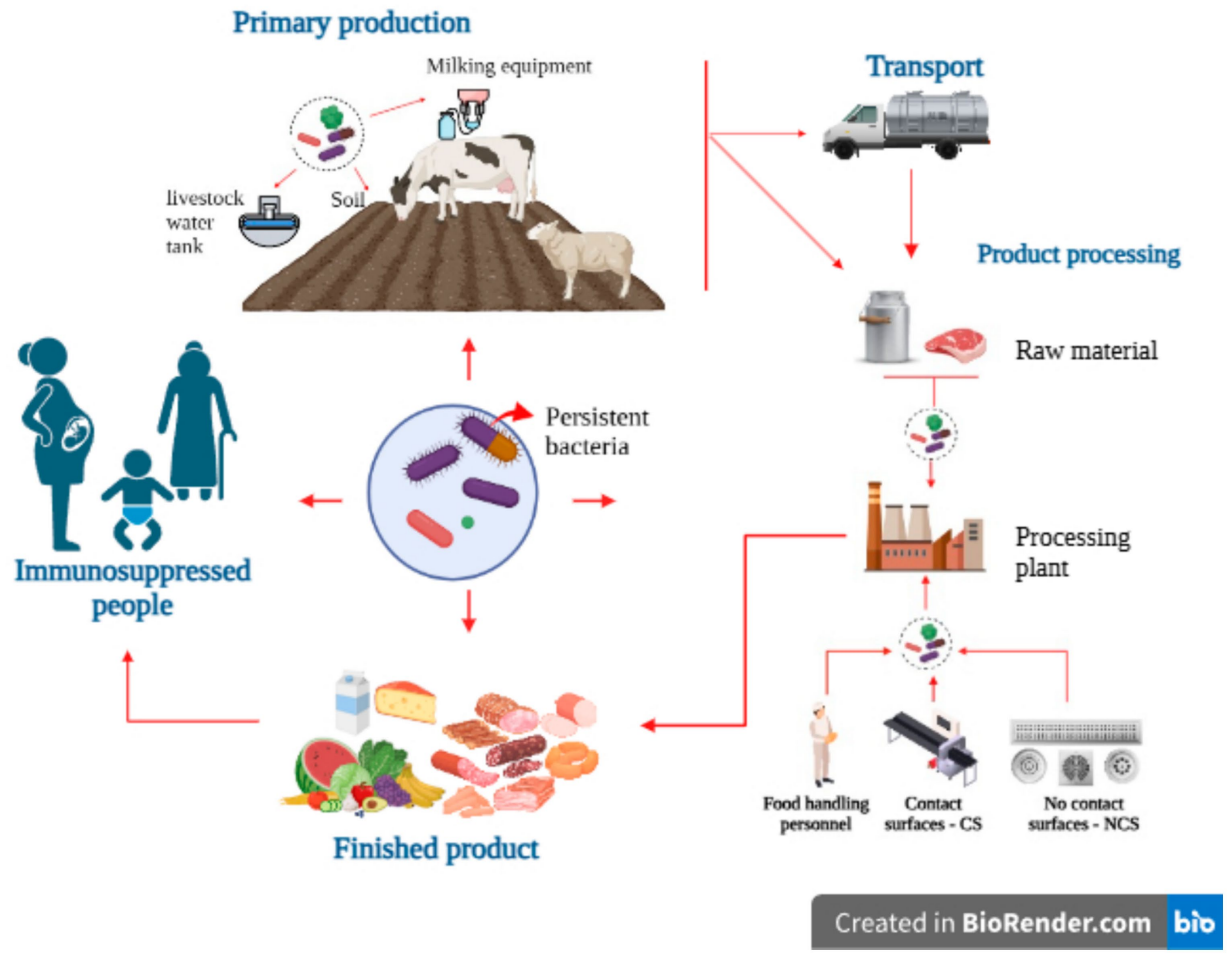
*Listeria monocytogenes* persistence pathways. Created in https://BioRender.com and some vector was designed by macrovector/Freepik: www.freepik.com.

Remarkably, the persistence of *L. monocytogenes* is a dynamic phenomenon, sustained by multiple factors that interact throughout the production and consumption chain (Figure 2). The combination of genetic adaptations, tolerance to adverse conditions and exploitation of ecological niches explains its recurrent presence in clinical environments as well as in industrial and food contexts. These considerations allow placing the problem in a broad context, in which persistence is not attributable to a single factor, but rather to the balance between the bacteria’s own capabilities and the opportunities offered by different environments.

### 3.4 Prospects for review

This exploratory review highlights that the persistence of *L. monocytogenes* in agri-food environments is a complex phenomenon, sustained by the interaction of genetic, ecological and technological factors. The information gathered demonstrates that a limited set of clonal complexes (CC1, CC2, CC4, CC6, CC9, CC31, CC87, CC121, CC321) is recurrently associated with chronic contamination that can last for years or even decades. The genomic stability observed in these lineages, even under conditions of extreme selective pressure such as cleaning, disinfection, nutrient scarcity or low temperatures, reveals a remarkable adaptive capacity that gives them competitive advantages over transient strains.

The use of high-resolution molecular methodologies (cgMLST, wgMLST, SNP/SNV analysis, and whole genome sequencing) has been crucial in differentiating between real episodes of persistence and simple reintroductions. However, the application of these tools remains uneven globally, with obvious limitations in low- and middle-income countries, creating critical gaps in the global management of the phenomenon and limiting comparisons between studies.

From an applied perspective, the systematic identification of resistance and tolerance genes, such as *qac*H, *bcr*ABC, *mdr*L, *cad*AC, as well as stress survival islands (SSI-1 and SSI-2) raises serious questions about the effectiveness of the sanitation protocols currently used in the industry. At the same time, the detection of truncated variants of inlA in some clones suggests divergent evolutionary trajectories: on the one hand, strains more suited to colonising food and industrial environments; on the other, a reduced capacity for invasion in the human host. This dualism poses an additional challenge in risk assessment and control policy design.

This systematic review adds value by integrating, under an exploratory approach, information usually fragmented across clinical, environmental, and industrial studies. Unlike previous reviews focused on a single area (e.g., human outbreaks, dairy products, or specific processing environments), this work comparatively articulates recent data on persistent clones, genetic determinants, and typing methodologies, highlighting convergences between different production sectors and geographical regions. This integration allows the global trends to be visualised and to highlight those clones that repeatedly appear as protagonists of long-term persistence, thus attempting to offer a broader and more useful overview for researchers, regulatory bodies and industry.

Looking ahead, it is essential to promote longitudinal surveillance programmes that combine standardised genomic typing with contextual information on facility design, hygiene practices and microecological characteristics. At the same time, there is an urgent need to consolidate international genomic repositories and agree on operational definitions of “persistence” that allow for comparative analysis between regions and production sectors. Finally, the persistence of *L. monocytogenes* must be addressed from an interdisciplinary approach, integrating microbiology, food science, public health, and regulation to generate innovative, sustainable, and effective intervention strategies to address a problem that transcends borders and puts global food safety under strain.

## 4 Conclusion

Analysis of the persistence of *L. monocytogenes* in human, animal, environmental, industrial and food contexts confirms that it is an intrinsically multifactorial phenomenon, resulting from the interaction between genetic, physiological, ecological and structural determinants. The remarkable biological plasticity of this bacterium allows it to adapt to heterogeneous selective pressures, which explains its continuous circulation between natural niches, agricultural systems, industrial environments and food matrices.

From a genomic and functional perspective, persistence cannot be attributed to a single trait or clone, but rather to sets of tolerance and adaptation mechanisms expressed in a context-dependent manner. In the human host, these mechanisms facilitate intestinal persistence and, in scenarios, progression to invasive forms, reinforcing the nature of *L. monocytogenes* as a pathogen capable of overcoming multiple physiological barriers.

In the animal and environmental setting, evidence shows that the bacterium remains silent in soil, water, manure and production systems, forming ecological reservoirs that sustain long-term clonal circulation. This dynamic locates persistence beyond a vision restricted to the industrial environment, integrating it into a broad ecological cycle in which animal reservoirs, environmental vectors and productive activities interact.

In processing plants, persistence occurs mainly as a structural and operational problem, where the convergence of bacterial factors and conditions specific to the facilities, such as drains, cracks and hard-to-reach equipment, favours chronic colonisation and recurrent recontamination. Thus, industrial environments not only act as reservoirs, but also as amplifiers of contamination towards the final products. In the case of ready-to-eat foods, the physiological characteristics of *L. monocytogenes* allow it to survive and multiply under adverse conditions, consolidating the food matrix as an active scenario for persistence, not merely as a vehicle for transmission.

Overall, the persistence of *L. monocytogenes* should be understood as the result of a dynamic network connecting human hosts, animals, natural environments, processing facilities, and food in a continuous cycle of adaptation and survival. This integrated view highlights the need to move beyond fragmented approaches and advance towards interpretations and strategies that simultaneously consider the genetic, ecological, and operational determinants involved. Finally, although certain factors associated with persistence are reported more frequently in the literature, the available evidence does not allow for the establishment of a universal hierarchy of influence, as their impact depends on the environmental and operational context in which the bacterium develops.

Overall, several crucial priorities will allow for advances in the understanding and management of *L. monocytogenes* persistence. These include the need to strengthen longitudinal surveillance approaches that allow for a more robust distinction between persistence and reintroduction, the development of harmonised operational definitions that integrate genomic, temporal and contextual criteria, and the reduction of existing gaps in the availability and comparability of data on a global scale. Addressing these priorities is fundamental to improving the interpretation of the phenomenon and avoiding fragmented responses based on partial evidence.

## Glossary

ADI: Arginine deiminase system
ALOA: Agar Listeria according to Ottaviani and Agosti
ANOVA: Analysis of variance
ATR: Acid Tolerance Response
BAC: Benzalkonium chloride
bp: Base pairs
CC: Clonal complex
CC1: Clonal Complex 1 – globally distributed; often linked to clinical cases and long-term circulation
CC2: Clonal Complex 2 – frequent in clinical and environmental isolates; persistent clusters reported
CC4: Clonal Complex 4 – associated with hypervirulent lineages and sustained transmission
CC6: Clonal Complex 6 – reported in food environments and clinical settings; persistence documented
CC8: Clonal Complex 8 – recovered in meat/processing environments; persistence reported
CC9: Clonal Complex 9 – common in meat and dairy processing; environmental persistence widely reported
CC31: Clonal Complex 31 – detected in pork sectors; disinfectant-tolerant and persistent
CC87: Clonal Complex 87 – retail/animal-linked clusters; long-term persistence in Asia/retail markets
CC121: Clonal Complex 121 – industry-adapted; often carries QAC tolerance determinants (e.g., qacH)
CC321: Clonal Complex 321 – prevalent QAC-tolerance locus (bcrABC) in US food environments
CFSAN: Centre for food safety and applied nutrition (FDA, USA)
CFU: Colony forming units
cgMLST: Core genome MultiLocus sequence typing
cm: Centimetre
CT4420: cgMLST Type 4,420 – reported in Brazil (ST9)
CT4429: cgMLST Type 4,429 – reported in Brazil (ST520)
DNA: Deoxyribonucleic acid
EFSA: European food safety authority
*emr*E: Efflux pump resistance gene (emrE)
FDA: Food and drug administration (United States)
GWAS: Genome-wide association studies
hqSNP: High-quality single nucleotide polymorphism
hqSNPs: High-quality single nucleotide polymorphisms
HPP: High-pressure processing
HT: Heat treatment
HT-29: Human colon epithelial cell line HT-29
kb: Kilobase (1,000 base pairs)
L1: Lineage I
L2: Lineage II
LIPI-3 / LIPI-4: Listeria Pathogenicity Island 3/4
LTA: Lipoteichoic acids
MALDI-TOF MS: Matrix-assisted laser desorption/ionisation time of flight mass spectrometry
MBC: Minimum bactericidal concentration
MIC: Minimum inhibitory concentration
MLST: MultiLocus sequence typing
MPa: Megapascal
MPN: Most probable number
MS: Mass spectrometry
nm: Nanometre (as in OD600 nm)
OD_600_: Optical density at 600 nm
PALCAM: Polymyxin–Acriflavine–Lithium Chloride–Ceftazidime–Aesculin–Mannitol agar
PCR: Polymerase chain reaction
PFGE: Pulsed-field gel electrophoresis
PMSCs: Premature stop codons
QAC: Quaternary ammonium compounds
RAST: Rapid annotation using subsystem technology
RTE: Ready-to-eat
ROS: Reactive oxygen species
SDS-PAGE: Sodium dodecyl sulphate–polyacrylamide gel electrophoresis
SID: Simpson’s index of diversity
SL9: Sublineage 9
SNPs: Single nucleotide polymorphisms
SNVs: Single nucleotide variants
SPAdes: St. Petersburg genome assembler
SSI-1/SSI-2: Stress survival islet 1/2st sequence type
ST9: Sequence Type 9 – founder of CC9, frequent in meat environments
ST31: Sequence Type 31 – member of CC31; found in pork-processing, disinfectant tolerant
ST121: Sequence Type 121 – member of CC121; often industry-adapted
ST520: Sequence Type 520 – reported in persistent Brazilian clusters (lineage I)
SSI: Statens Serum Institut
TOF: Time of flight
WGS: Whole genome sequencing
wgMLST: Whole genome MultiLocus sequence typing
WTA: Wall teichoic acids
