# Supplementary material for: Persistence of Listeria monocytogenes: an integrative narrative review
Source: Front Microbiol. 2026 Jan 20;17:1721836. doi: 10.3389/fmicb.2026.1721836 (PMC12864391; doi:10.3389/fmicb.2026.1721836)
Supplement: Supplementary file 1 [file Table_1.docx]

**Supplementary Material SM1**

MS1. Techniques employed in persistence studies of Listeria monocytogenes.

| **Technique** | **Brief explanation** | **Ref.** |
| --- | --- | --- |
| PCR (Polymerase Chain Reaction) | Amplifies specific DNA fragments to confirm the identity of *L. monocytogenes* or detect virulence genes (*inl*A, *inl*B), resistance genes (*bcr*ABC, *qac*H), or stress-related loci. It is the first step before advanced genomic analyses. | (Lundén et al., 2003; Cherifi et al., 2020; Demaitre et al., 2021) |
| Multiplex PCR | Extension of PCR allowing simultaneous amplification of several markers in a single reaction, useful for rapid serogroup determination. | (Demaitre, et al. 2021) |
| PFGE (Pulsed-Field Gel Electrophoresis) | Separates large DNA fragments generated by rare-cutting restriction enzymes. Produces banding profiles serving as strain fingerprints. For decades, it was the gold standard for persistence typing. | (Lundén et al., 2000; Cardozo-Bernal et al., 2013; Wang et al., 2015; Taylor et al., 2019; Cherifi, et al. 2020; Demaitre, et al. 2021) |
| MLST / cgMLST / wgMLST | Based on sequencing housekeeping genes (MLST) or hundreds/thousands of loci (cgMLST/wgMLST). Classifies isolates into STs and CCs, enabling long-term tracking of persistent clones in facilities or across countries. | (Pasquali et al., 2018; Sullivan et al., 2022; Ikhimiukor et al., 2024; van de Merwe et al., 2024) |
| WGS (Whole Genome Sequencing) | Provides complete genomic information, distinguishing between reintroductions and true persistence. Detects SNPs, plasmids, prophages, and resistance islands. | (Daeschel et al., 2022; Sullivan, et al. 2022; Wang et al., 2022; Ikhimiukor, et al. 2024) |
| SNP / SNV Analysis | Genome-wide comparison at single-nucleotide resolution. Persistence is often defined by thresholds (≤10–20 SNPs) classifying isolates as the same clone. | (Sullivan, et al. 2022; Ikhimiukor, et al. 2024; van de Merwe, et al. 2024) |
| GWAS | Associates genomic features with phenotypes such as disinfectant resistance or persistence. Requires large datasets of isolates. | (Daeschel, et al. 2022) |
| Prokka / RAST Annotation | Bioinformatics pipelines annotating draft genomes, predicting genes, stress survival islands (SSI-1, SSI-2), and resistance cassettes. Links gene content to persistence traits. | (Daeschel, et al. 2022; Domingues et al., 2025) |
| SPAdes Genome Assembler | Bioinformatics assembler reconstructing draft genomes from sequencing reads, often used prior to annotation or comparative genomics. | (Ikhimiukor, et al. 2024) |
| Prophage detection | Identifies prophages integrated into hotspots such as *com*K. These elements disrupt bacterial competence or functions and contribute to persistent behaviour. | (Wang, et al. 2022) |
| Plasmid profiling | Detection and characterisation of plasmids (e.g., *p*LM1686, *p*LM1692) carrying resistance genes (cadmium, QAC, stress tolerance). | (Wang, et al. 2022) |
| Most Probable Number (MPN) | Statistical culture-based method estimating viable bacteria from serial dilutions and growth-positive tubes. In persistence studies, MPN quantifies Listeria in manure or compost at low levels. | (Grewal et al., 2007) |
| Selective culture media (Oxford, PALCAM agar) | Selective agars containing antibiotics and chromogenic substrates suppress competing flora while enabling Listeria identification. Essential for recovering low-level environmental isolates. | (Grewal, et al. 2007; Cherifi, et al. 2020) |
| Biofilm assays (crystal violet staining) | Quantify biofilms by staining biomass adhered to abiotic surfaces with crystal violet and measuring absorbance. Persistent isolates often form stronger biofilms. | (Wang, et al. 2015; Rohilla et al., 2024) |
| Adhesion assays | Short-term tests measuring attachment to plastic or stainless-steel surfaces. Early adhesion is a key factor for persistence in industrial environments. | (Wang, et al. 2015) |
| Cold/acid/salt/osmotic stress assays | Growth under stressful conditions (e.g., 10 % (w/v) NaCl, pH 5.2, refrigeration) evaluates phenotypic tolerance of persistent versus sporadic isolates. | (Taylor, et al. 2019; Domingues, et al. 2025) |
| Cell line infection assays (HT-29) | Human colon epithelial cell line HT-29 used to measure invasion and intracellular survival. Links persistence phenotypes to virulence potential. | (Ikhimiukor, et al. 2024) |
| Protein profiling (SDS-PAGE) | Separates proteins by size, allowing analysis of stress protein expression or biofilm-associated proteins. | (Ortiz et al., 2016; Domingues, et al. 2025) |
| Growth kinetics (OD_600_) | Monitoring OD_600 nm_ in microplate readers measures bacterial growth under different nutrients or stresses. | (Taylor, et al. 2019) |
| Minimum Bactericidal Concentration (MBC) | Lowest concentration of a chemical (e.g., BAC) that kills ≥99.9% of the population. Applied to persistent isolates to test disinfectant tolerance. | (Cherifi, et al. 2020) |
| Minimum Inhibitory Concentration (MIC) | Lowest concentration of an antimicrobial that prevents visible growth. Often measured alongside MBC in disinfectant studies. | (Wang, et al. 2015; Daeschel, et al. 2022) |
| Efflux pump inhibitor assays | Chemical inhibitors used to block efflux activity and confirm whether genes such as *bcr*ABC or *qac*H are directly responsible for tolerance. | (Daeschel, et al. 2022) |
| Metal tolerance assays | Expose isolates to increasing concentrations of metals (cadmium, arsenic, copper, zinc) to confirm resistance and link with genes (*cad*AC). | (Wang, et al. 2022; Ikhimiukor, et al. 2024) |
| High Pressure Processing (HPP, 400–600 MPa) | Food safety intervention applying hydrostatic pressure to inactivate pathogens. Tested on persistent lineages to evaluate survival differences between lineages I and II. | (van de Merwe, et al. 2024) |
| Pan-genome analysis | Defines the 'core' genome shared by all isolates and the 'accessory' genes unique to subsets. Identifies traits enriched in persistent clones. | (Pasquali, et al. 2018; Ikhimiukor, et al. 2024) |
| Phylogenetic reconstruction | Based on SNPs or MLST, reconstructs phylogenetic trees to trace relationships and distinguish persistence from reintroductions. | (Sullivan, et al. 2022; Ikhimiukor, et al. 2024) |
| Simpson’s Index of Diversity (SID) | Ecological diversity index applied to genotyping data to measure discriminatory power of typing methods or clonal diversity. | (Latorre et al., 2011) |
| MALDI-TOF MS | Matrix-Assisted Laser Desorption/Ionisation Time-of-Flight Mass Spectrometry identifies bacteria by protein spectra. Less discriminatory than WGS but useful for preliminary characterisation. | (Ortiz, et al. 2016) |

**References**

Cardozo-Bernal ÁM, Poutou-Piñales RA, Carrascal-Camacho AK, Ramón LF, Zambrano DC. (2013). Electroforesis en Gel de Campo Pulsado (PFGE) para la diferenciación molecular de *Listeria monocytogenes*. *Univ. Sci*. 18:203-222. <http://doi.org/10.11144/Javeriana.SC18-2.egcp>.

Cherifi T, Arsenault J, Pagotto F, Quessy S, Cote JC, Neira K, Fournaise S, Bekal S, Fravalo P. (2020). Distribution, diversity and persistence of *Listeria monocytogenes* in swine slaughterhouses and their association with food and human listeriosis strains. *Plos One*. 15:e0236807. <https://doi.org/10.1371/journal.pone.0236807>.

Daeschel D, Pettengill JB, Wang Y, Chen Y, Allard M, Snyder AB. (2022). Genomic analysis of *Listeria monocytogenes* from US food processing environments reveals a high prevalence of QAC efflux genes but limited evidence of their contribution to environmental persistence. *BMC Genom*. 23:488. <http://doi.org/10.1186/s12864-022-08695-2>.

Demaitre N, Rasschaert G, De Zutter L, Geeraerd A, De Reu K. (2021). Genetic *Listeria monocytogenes* types in the pork processing plant environment: from occasional introduction to plausible persistence in harborage sites. *Pathog*. 10:717. <http://doi.org/10.3390/pathogens10060717>.

Domingues CPF, Almeida G, Teixeira P, Nogueira T. (2025). Persistence of *Listeria monocytogenes* in food processing environments: challenges and future directions. *Acad. Mol. Biol. Genom.* 2:1-6. <http://doi.org/10.20935/AcadMolBioGen7715>.

Grewal S, Sreevatsan S, Michel FC. (2007). Persistence of Listeria and Salmonella during swine manure treatment. *Comp. Sci. Utiliz*. 15:53-62. <http://doi.org/10.1080/1065657x.2007.10702311>.

Ikhimiukor OO, Mingle L, Wirth SE, Mendez-Vallellanes DV, Hoyt H, Musser KA, Wolfgang WJ, Andam CP. (2024). Long-term persistence of diverse clones shapes the transmission landscape of invasive *Listeria monocytogenes*. *Genom. Med*. 16:109. <http://doi.org/10.1186/s13073-024-01379-4>.

Latorre AA, Van Kessel JA, Karns JS, Zurakowski MJ, Pradhan AK, Boor KJ, Adolph E, Sukhnanand S, Schukken YH. (2011). Increased *in vitro* adherence and on-farm persistence of predominant and persistent *Listeria monocytogenes* strains in the milking system. *Appl. Environ. Microbiol*. 77:3676-3684. <http://doi.org/10.1128/AEM.02441-10>.

Lundén JM, Autio TJ, Markkula A, Hellstrom S, Korkeala HJ. (2003). Adaptive and cross-adaptive responses of persistent and non-persistent *Listeria monocytogenes* strains to disinfectants. *Int. J. Food Microbiol*. 82:265-272.

Lundén JM, Miettinen MK, Autio T, J., Korkeala HJ. (2000). Persistent *Listeria monocytogenes* strains show enhanced adherence to food contact surface after short contact times. *J. Food Prot*. 63:1204-1207.

Ortiz S, Lopez-Alonso V, Rodriguez P, Martinez-Suarez JV. (2016). The connection between persistent, disinfectant-resistant *Listeria monocytogenes* strains from two geographically separate iberian pork processing plants: Evidence from comparative genome analysis. *Appl. Environ. Microbiol*. 82:308-317. <http://doi.org/10.1128/AEM.02824-15>.

Pasquali F, Palma F, Guillier L, Lucchi A, De Cesare A, Manfreda G. (2018). *Listeria monocytogenes* sequence types 121 and 14 repeatedly isolated within one year of sampling in a rabbit meat processing plant: Persistence and ecophysiology. *Front. Microbiol*. 9:596. <http://doi.org/10.3389/fmicb.2018.00596>.

Rohilla A, Kumar V, Ahire JJ. (2024). Unveiling the persistent threat: recent insights into *Listeria monocytogenes* adaptation, biofilm formation, and pathogenicity in foodborne infections. *J. Food Sci. Technol*. 61:1428-1438. <http://doi.org/10.1007/s13197-023-05918-6>.

Sullivan G, Orsi RH, Estrada E, Strawn L, Wiedmanna M. (2022). Whole-Genome Sequencing-Based characterization of Listeria isolates from produce packinghouses and fresh-cut facilities suggests both persistence and reintroduction of fully virulent *L. monocytogenes*. *Appl. Environ. Microbiol*. 88:1-18. <http://doi.org/10.1128/aem.01177-22>.

Taylor AJ, Stasiewicz MJ. (2019). Persistent and sporadic *Listeria monocytogenes* strains do not differ when growing at 37 degrees C, in planktonic state, under different food associated stresses or energy sources. *BMC Microbiol*. 19:257. <http://doi.org/10.1186/s12866-019-1631-3>.

van de Merwe C, Simpson DJ, Qiao N, Otto SJG, Kovacevic J, Gänzle MG, McMullen LM. (2024). Is the persistence of *Listeria monocytogenes i*n food processing facilities and its resistance to pathogen intervention linked to its phylogeny? *Appl. Environ. Microbiol*. 90:1-18. <http://doi.org/10.1128/aem.00861-24>.

Wang J, Ray AJ, Hammons SR, Oliver HF. (2015). Persistent and transient *Listeria monocytogenes* strains from retail deli environments vary in their ability to adhere and form biofilms and rarely have *inl*A premature stop codons. *Foodborne Pathog. Dis*. 12:151-158. <http://dx.doi.org/10.1089/fpd.2014.1837>.

Wang Y, Luo L, Ji S, Li Q, Wang H, Zhang Z, Mao P, Sun H, Li L, Wang Y, et al. (2022). Dissecting *Listeria monocytogenes* persistent contamination in a retail market using Whole-Genome Sequencing. *Microbiol. Spect*. 10:1-12. <http://doi.org/10.1128/spectrum.00185-22>.
